# Supplementary material for: DNA satellite and chromatin organization at house mouse centromeres and pericentromeres
Source: bioRxiv. 2023 Jul 19:2023.07.18.549612. Preprint. [Version 1] doi: 10.1101/2023.07.18.549612 (PMC10370071; doi:10.1101/2023.07.18.549612)
Supplement: Supplement 1 [file media-1.pdf]

SUPPLEMENTARY FIGURES

A) Read length distribution

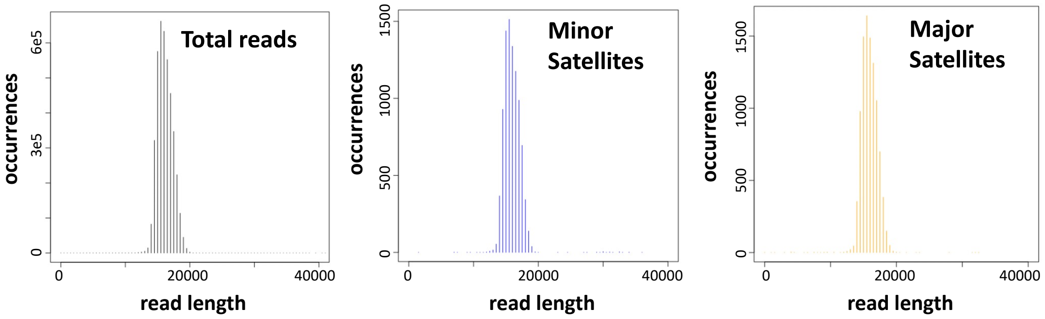

B) MiSat arrays

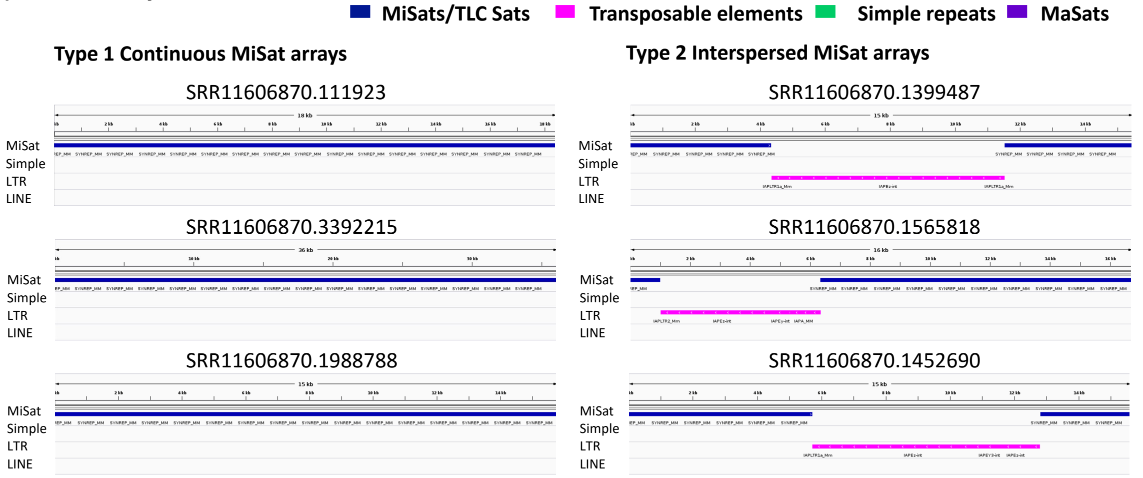

C) Centromere and telomere junctions

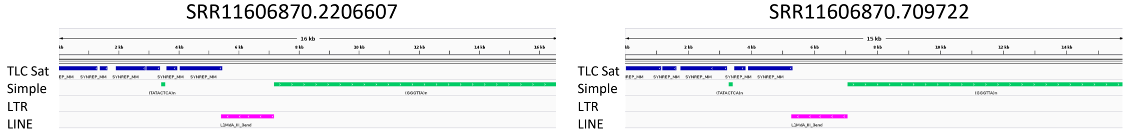

D) MaSat arrays

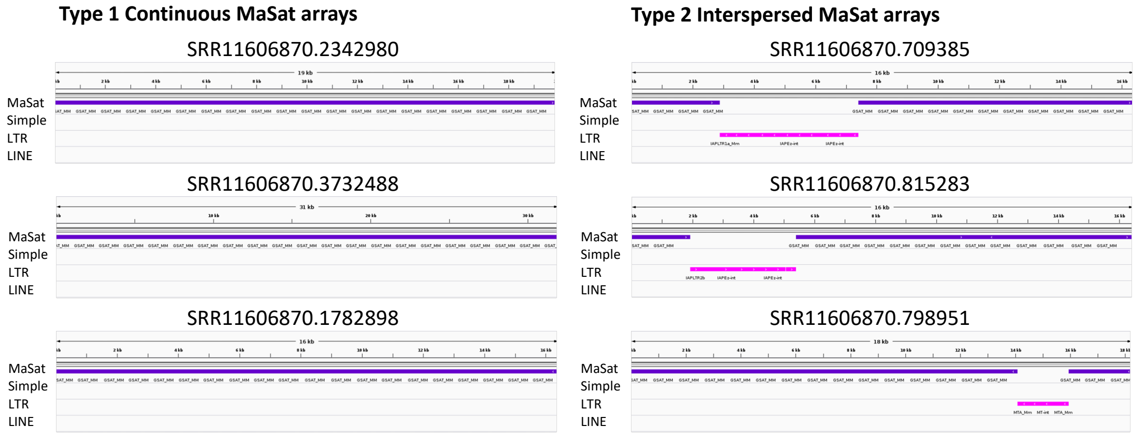

Supplementary Figure 1. A) Read length distribution in the LRS data analyzed in this study. Detailed organization of sample LRS reads with B) MiSat arrays, C) Centromere-Telomere junctions, and D) and MaSat arrays.

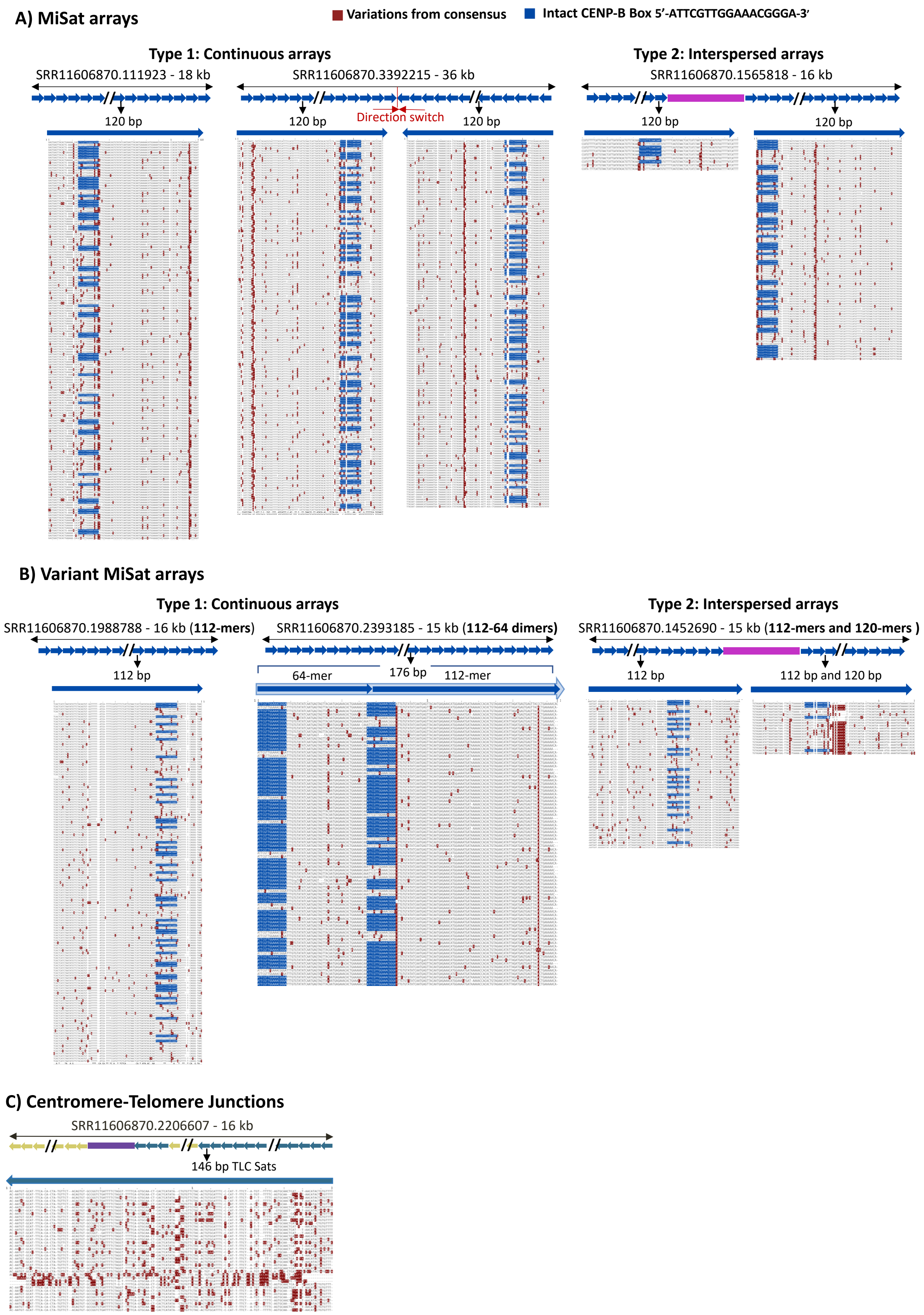
A) MiSat arrays
Variations from consensus
Intact CENP-B Box 5'-ATTCGTTGGAAACGGGA-3'
Type 1: Continuous arrays
SRR11606870.111923 - 18 kb
SRR11606870.3392215 - 36 kb
120 bp
Direction switch
Type 2: Interspersed arrays
SRR11606870.1565818 - 16 kb
B) Variant MiSat arrays
SRR11606870.1988788 - 16 kb (112-mers)
SRR11606870.2393185 - 15 kb (112-64 dimers)
112 bp
64-mer
176 bp
112-mer
SRR11606870.1452690 - 15 kb (112-mers and 120-mers)
112 bp and 120 bp
C) Centromere-Telomere Junctions
SRR11606870.2206607 - 16 kb
146 bp TLC Sats

Supplementary Figure 2. Full alignments from MiSat and telomere-centromere junction TLC arrays.

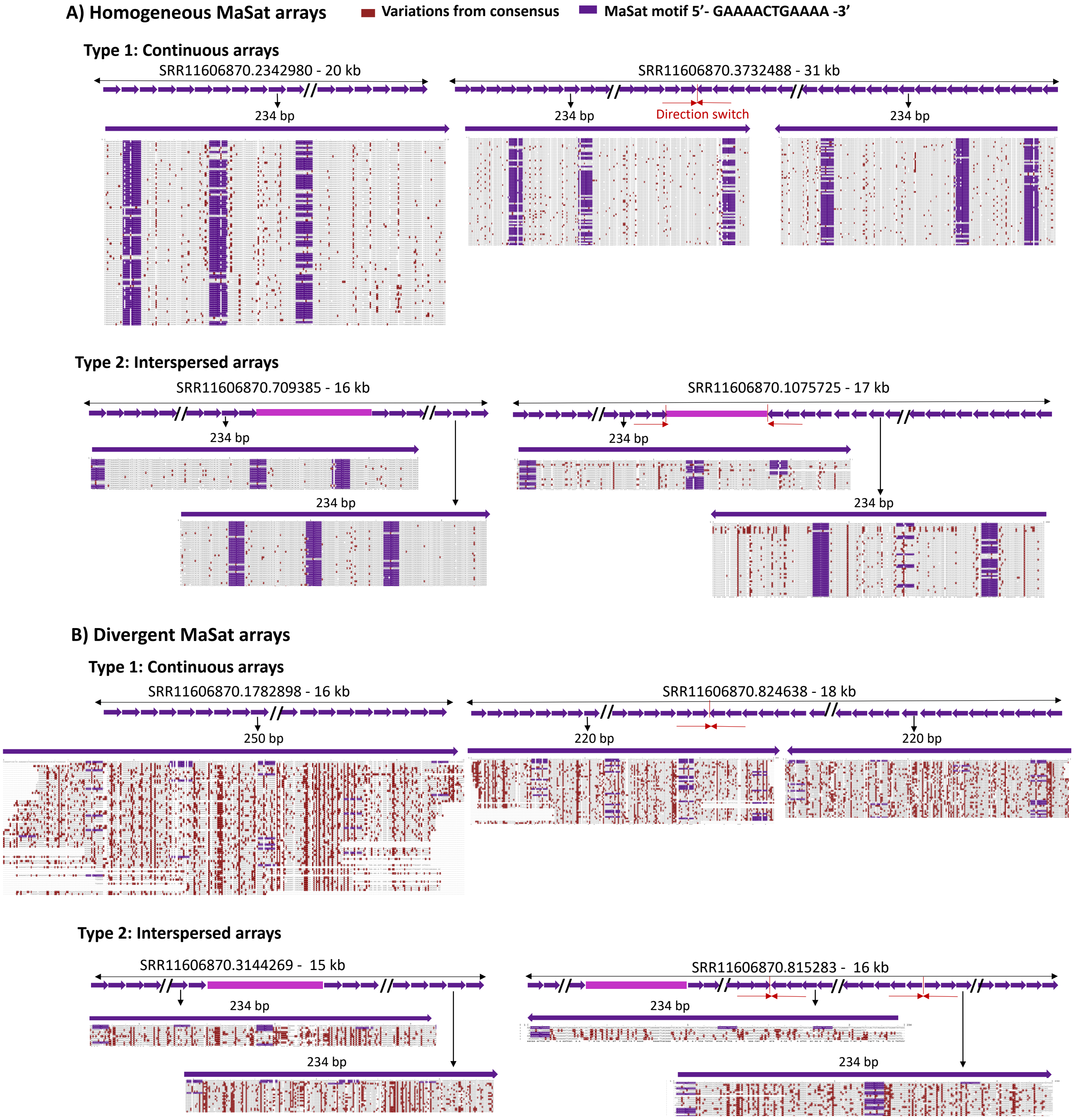

Supplementary Figure 3. Full alignments from MaSat arrays.

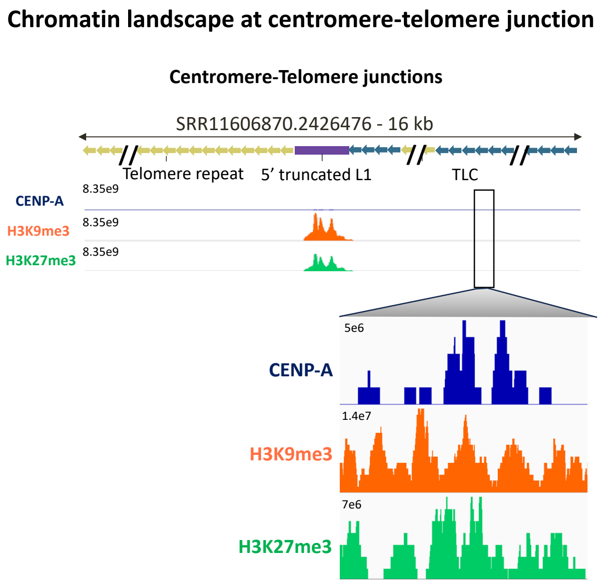

Supplementary Figure 4. CENP-A, H3K9me3, and H3K27me3 chromatin profiles at centromere-telomere junction arrays including TLC arrays.
